# Voclosporin shows protective effect and intestinal barrier enforcement in experimental colitis

**DOI:** 10.3389/fmed.2026.1750826

**Published:** 2026-02-02

**Authors:** M. Gabel, A. Knauss, Y. Liu, M. Mohamed Abdou, C. Kaufmann, L. Loges, M. Stürzl, S. Schürmann, M. J. Waldner, M. F. Neurath, B. Weigmann

**Affiliations:** 1 Department of Medicine 1, University Hospital Erlangen, Erlangen, Germany; 2 Division of Molecular and Experimental Surgery, Department of Surgery, Translational Research Center, Friedrich-Alexander University Erlangen-Nürnberg and University Hospital Erlangen, Erlangen, Germany; 3 Institute of Medical Biotechnology, Friedrich-Alexander University Erlangen-Nürnberg, Erlangen, Germany; 4 Deutsches Zentrum Immuntherapie (DZI), Erlangen, Germany; 5 FAU Profile Center Immunomedicine, Erlangen, Germany

**Keywords:** calcineurin inhibitor, experimental colitis, IBD, treatment, Voclosporin

## Abstract

The treatment of inflammatory bowel disease (IBD) is still challenging. Therefore, it is crucial not only to develop new drugs specifically targeting IBD but also to evaluate the application and efficacy of already established pharmaceuticals used for related disorders. A promising new candidate is Voclosporin (Voc), a recently approved drug for lupus nephritis. In this study, we aimed to further elucidate the efficiency and the molecular mechanism of action of Voclosporin in comparison with its analogon cyclosporine A (CsA). Using an experimental colitis model and human PBMCs, we performed a comprehensive analysis including mini-endoscopy, histopathology, multi-photon endomicroscopy (MPEM), immunofluorescence staining, flow cytometry, and cytokine secretion profiling of murine lamina propria mononuclear cells (LPMCs). Treatment with Voc or CsA improved colitis-associated weight loss and reduced intestinal inflammation as assessed by endoscopy and histopathological stainings. Treatment with Voclosporin led to a significant increase of the barrier-strengthening protein claudin 3 in the colon of mice with experimentally induced colitis. Furthermore, treatment of stimulated human-derived PBMCs from healthy controls with Voclosporin and CsA inhibited the activation of IL2-inducible tyrosine kinase ITK, a known trigger of inflammation in IBD. These results further support the potential of Voclosporin as a promising therapeutic strategy for the treatment of acute intestinal inflammation.

## Graphical abstract

**Figure:**
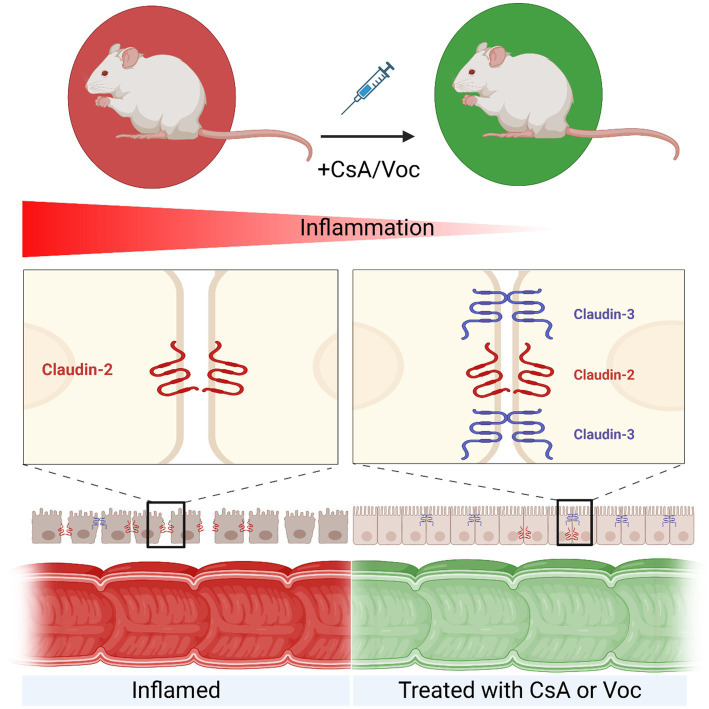
Voclosporin and CsA have a protective effect and strengthen the intestinal barrier in experimental colitis, with Voclosporin having a more pronounced effect. Treatment with CsA or Voclosporin reduces inflammation in the intestine. At the molecular level, treatment with calcineurin inhibitors leads to an increase in claudin 3, which helps to restore the intestinal barrier, while the amount of pore-forming claudin 2 remains unchanged. Created in BioRender. Knauß, A. (2026) https://BioRender.com/l1hkpqy.

## Introduction

Inflammatory bowel disease (IBD) and its two predominant subforms, Crohn’s disease (CD) and ulcerative colitis (UC), signify a pervasive global issue. Given that inflammatory bowel disease affects approximately 7 million individuals worldwide, continued investment in research on this disorder remains essential (1). Cyclosporine A (CsA), a calcineurin inhibitor, arose 30 years ago and still displays an effective alternative to corticosteroids for the treatment of steroid-refractory UC patients (2, 3). However, a large patient group remains who does not respond properly to medical therapy with CsA and the general toxicity of CsA as well as the need for therapeutic drug monitoring should not be disregarded (4, 5). A promising novel therapeutic agent from the family of calcineurin inhibitors is Voclosporin. It was approved by the EMA in 2022 based on the data achieved by the AURORA 1 trial for the treatment of lupus nephritis (6). Voclosporin represents a potent analogon of CsA, wherein a specific side chain is chemically modified. This modification improves the efficacy of calcineurin inhibition and, at the same time, the tolerability in various immunological diseases like psoriasis, organ transplantation, uveitis, and lupus nephritis (7). Studies showed not only the safety and efficacy of a long-term treatment in patients with lupus nephritis but also indicated a potential drug option for patients with acute severe steroid-refractory colitis (8, 9). Lechner et al. showed that the IL2-inducible tyrosine kinase (ITK) is involved in the pathogenesis of autoimmune diseases like ulcerative colitis (10, 11). ITK has been identified and characterized as a mediator of T cell activation downstream of T cell receptor signaling (12–16). Triggering the T cell receptor leads to the activation of the intracellular Src family kinase Lck that subsequently induces the phosphorylation of the tyrosine kinase Zap70 that further initiates the activation of ITK. The suppression of ITK function has been shown to alter T cell activation, cytokine production and can improve experimental colitis in mice. Additionally, ITK has been termed to be an important player that drives inflammation in IBD (11, 17–20). Moreover, our group previously published that CsA treatment targets and modulates ITK in T cells displayed by reduced phosphorylation and activation, respectively (11). Lindemann et al. showed for the first time a protective effect of Voclosporin in an acute dextran sodium sulfate-induced model of colitis in mice (9). In this study, we first aimed to assess and confirm the efficacy and effectiveness of Voclosporin in a different murine model of acute colitis. Therefore, we induced colonic inflammation with oxazolone and either treated mice with CsA, Voclosporin, or a solvent control. We used *in vivo* analysis to evaluate inflammation and further investigated the molecular mechanisms related to ITK accessing flow cytometry, immunofluorescence stainings, and cytokine analysis. Intestinal vascular permeability was tested using multiphoton endomicroscopy, and barrier function was assessed based on measurements of claudin 2/3 molecules. In a second approach, we examined the effect of the pharmacological treatment by CsA or Voclosporin on ITK activation in stimulated human PBMCs. Due to the similar structure of CsA and Voclosporin we hypothesized a similar or reinforced impact of Voclosporin on the colitis outcome and ITK inhibition.

## Results

### Cyclosporine A and Voclosporin treatment ameliorate acute oxazolone-induced colitis

**Figure 1:**
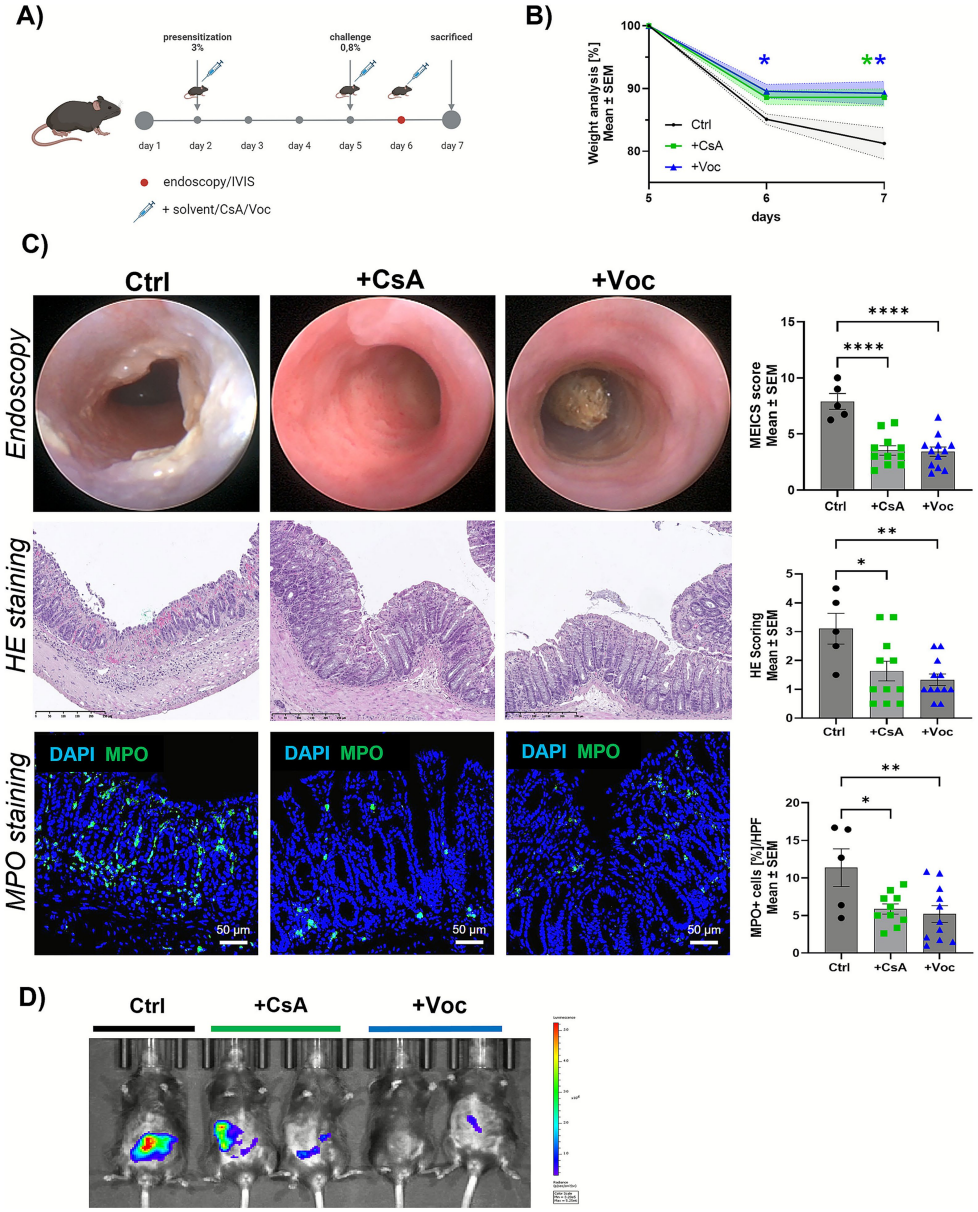
Cyclosporine A and Voclosporin treatment ameliorate acute oxazolone-induced colitis in mice. **(A)** Experimental setup of acute oxazolone colitis ± CsA/Voc treatment. **(B)** Weight development and course—day 1 and 2 after oxazolone challenge representing day 6 and 7 of the experiment (*n* = 5–12). **(C)** From top to bottom: endoscopic imaging and corresponding MEICs scoring (*n* = 5–12); HE staining of paraffin embedded colon samples and corresponding scoring; IF staining of MPO in colonic cryo sections and percentage of MPO+ cells (*n* = 5–11). **(D)** *In vivo* MPO imaging. Mean values ± SEM of two individual experiments are shown (*p < 0.05, **p < 0.01, ****p < 0.0001). **(A)** Created in BioRender. Knauß, A. (2026) https://BioRender.com/0tv0xjj.

To test the efficacy of Voclosporin in colitis and compare effects with the already established drug cyclosporine A, BL6 mice were challenged with oxazolone to induce acute severe colitis. On day 2, 5, and 6, we treated the mice with either 10 mg/kg CsA, Voclosporin, or a solvent control. We used mini-endoscopy imaging and IVIS imaging to evaluate the inflammation in the colon *in vivo* (Figure 1A). Mice treated with the solvent control showed drastic weight loss on day 6 (~15% weight loss) and 7 (~19% weight loss), representing the 1st and 2nd day after acute colitis induction. The application of Voclosporin decreased the weight loss significantly from the 1st day after the challenge (~10% weight loss on days 6 and 7), whereas CsA treatment reached significance on day 7 (~12% weight loss) (Figure 1B, Supplementary Figure 1). Both CsA and Voclosporin ameliorated colitis significantly, as shown by endoscopic imaging and the corresponding MEICs scoring (Figure 1C). Here, the analysis of parameters such as translucency, granularity, fibrin development, vascularity, and stool consistency collectively resulted in an improved colitis score. Evaluation of inflammation was also performed using HE staining and scoring as well as immunofluorescence staining of myeloperoxidase (MPO)-positive cells in the colon. Treatment with both calcineurin inhibitors resulted in a significant improvement of the consulted parameters, as the control group showed exacerbated tissue damage indicated by marked inflammatory cell infiltration, erosive tissue architecture, and increased MPO activity (Figure 1C). Moreover, displaying the *in vivo* activity of MPO via IVIS imaging underlined the effectiveness of CsA and Voclosporin in preventing a severe colitis course presented by downsized signal areas and minimized signal intensities (Figure 1D). Similar results were obtained when CsA and Voclosporin were administered in a preventive setting, where the drugs were given prior to the induction of colitis (Supplementary Figure 2).

### Cyclosporine A and Voclosporin suppress various T helper cell cytokines and mitigate the expression of intracellular kinases

**Figure 2:**
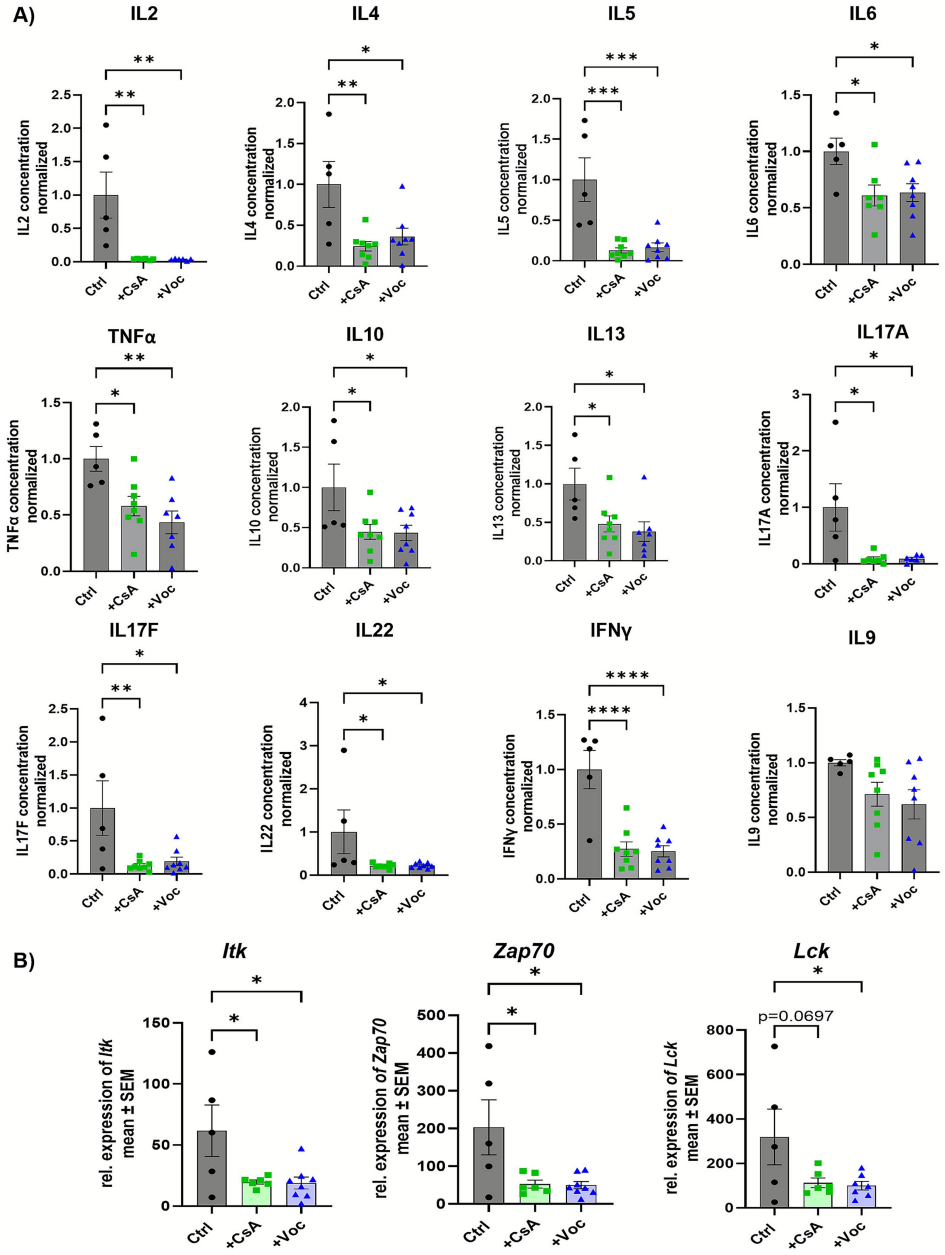
Cyclosporine A as well as Voclosporin decrease the production of various cytokines and lower the transcript expression of *Itk*, *Lck*, and *Zap70* in LPMCs. **(A)** Cytokine secretion and **(B)** transcript expression of *Itk*, *Lck*, and *Zap70* of cultured murine LPMCs derived from mice with induced colitis treated with solvent, CsA, or Voc (*n* = 5–8). LPMCs were isolated from murine colonic tissue, stimulated with αCD3/αCD28, and cultured for 48 h. The concentration of IL2, IL4, IL5, IL6, IL9, IL10, IL13, IL17A, IL17F, IL22, IFNγ, and TNFα in the supernatant was determined using a bead-based assay. Mean values ± SEM of two individual experiments are shown (*p < 0.05, **p < 0.01, ***p < 0.001, ****p < 0.0001).

Colonic LPMCs were isolated on the second day after initiating colitis from mice treated with solvent control, CsA, or Voc. The cells were stimulated with *α*-CD3/CD28 antibodies and cultured for 48 h without additional calcineurin inhibitor administration. Subsequently, the supernatant was used for cytokine analysis and cells for studying gene expression characteristics. Treatment with both CsA and Voclosporin significantly suppressed the release of IL2, IL4, IL5, IL6, IL10, IL13, IL17A, IL17F, IL22, IFNγ, and TNFα from cultured LPMCs (Figure 2A). Since CsA was proven to decrease phosphorylation of the intracellular kinase Itk (11), we investigated whether Voclosporin and CsA result in similar effects on transcript levels. Both calcineurin inhibitors equally diminished the relative expression of Itk in cultured LPMCs compared to the control group. Moreover, the expression of the upstream kinase Zap70 was significantly reduced following treatment with CsA or Voclosporin. Additionally, lower levels of Lck were detected in inhibitor-treated groups; however, in the case of CsA it did not reach statistical significance (Figure 2B). Taken together, these results suggest equivalent efficaciousness and potency regarding suppressing lymphocyte activity and cytokine responses.

### Colonic vascular permeability imaging revealed the potent efficiency of Cyclosporine A and Voclosporin in inhibiting vascular leakage in oxazolone-induced colitis murine models

**Figure 3:**
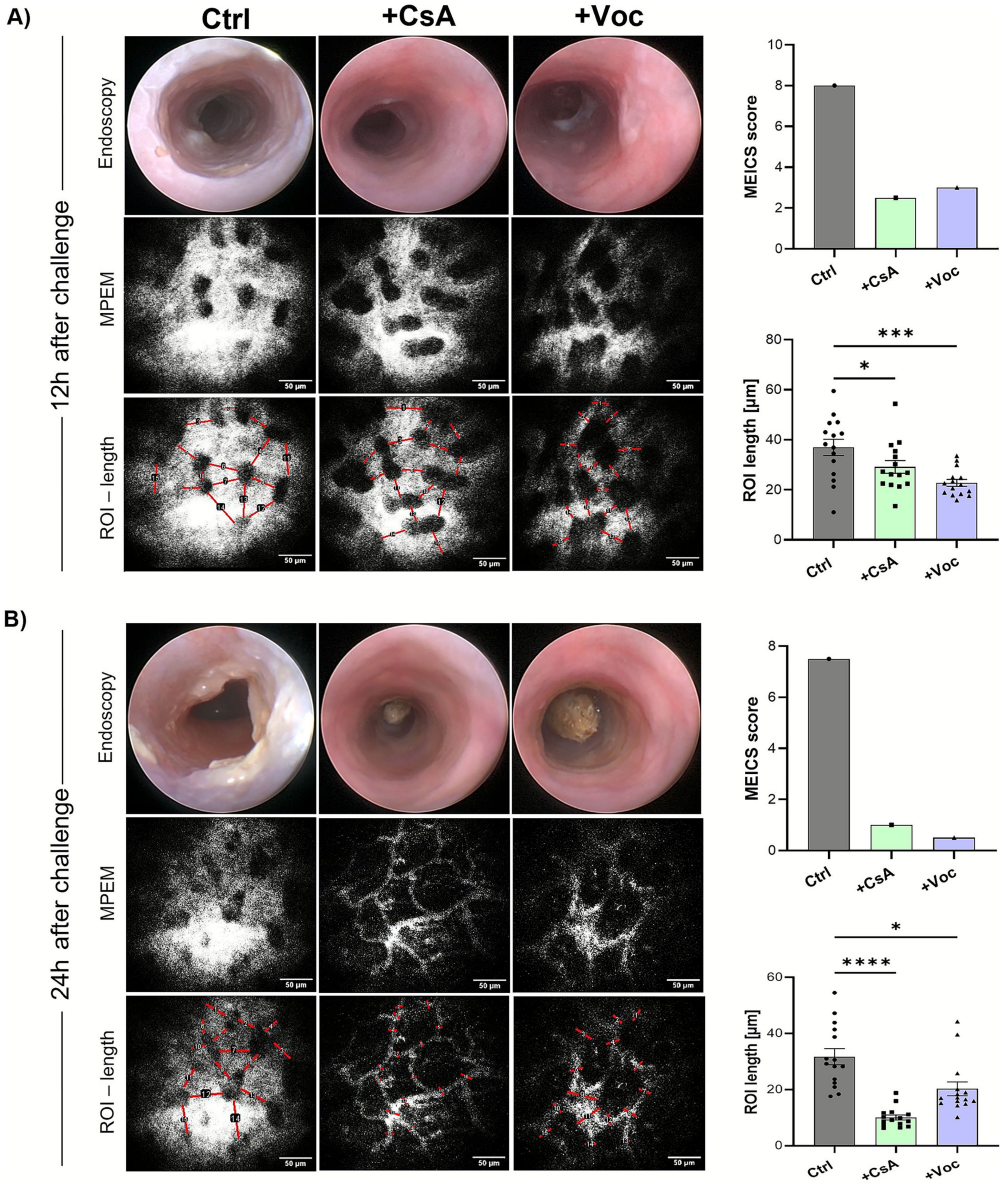
*In vivo* colonic vascular permeability imaging revealed the potent efficiency of cyclosporine A and Voclosporin in inhibiting the vascular leakage in oxazolone-induced colitis murine models. Oxazolone ± CsA/Voc-treated mice colons were longitudinally assessed at 12 h **(A)** and 24 h **(B)** following the oxazolone challenge for vascular permeability changes via MPEM (bottom panels; representative images for each treatment) and endoscopy (upper panels; representative images for each treatment condition) of the distal colons. Scale bars = 50 μm.

To further validate the efficiency of CsA and Voclosporin in mitigating oxazolone-induced colitis, we utilized a recently established technique that detects the changes in vascular permeability with high sensitivity and employs it as a precise marker of inflammation in murine distal colons [method adapted from Kreiß et al. (21)]. The oxazolone-challenged mice were either treated with CsA, Voclosporin, or solvent control. 12 h and 24 h after the oxazolone challenge, the mice were injected with 70 kDa FITC-dextran, serving as a contrast agent, followed by the colonic vascular permeability imaging using the MPEM (Figures 3A,B, bottom panels). Afterwards, they underwent endoscopy for the detection of macroscopic signs of inflammation in the colonic mucosa (Figures 3A,B, upper panels). 12 h after colitis induction, *in vivo* measurements of vascular permeability revealed a marked inflammatory response across all treated groups, as evidenced by the substantial extravasation of fluorescently labeled dextran molecules (Figure 3A, bottom panels). However, calcineurin-inhibitor-treated mice displayed significantly diminished vascular permeability compared to the control group, quantified by measuring the ROI length of the FITC dextran that has leaked out of the vessels. Meanwhile, the endoscopy imaging of the same mice showed a high degree of mucosal inflammation in solvent control-treated mice and, to a substantial lesser extent, in CsA and Voclosporin-treated ones (Figure 3A, upper panels). Strikingly, the MPEM imaging at 24 h following the oxazolone challenge of the same set of mice showed a significant reduction of vascular permeability in the mice treated with CsA or Voclosporin (Figure 3B, bottom panels), while elevated vascular leakage remained massive in the solvent control treated group. Consistent with this observation, endoscopic analysis revealed an almost complete absence of macroscopic inflammatory signs in the colonic mucosa of mice treated with CsA or Voclosporin (Figure 3B, upper panels). In contrast, mice treated with the solvent control exhibited marked fibrin accumulation and mucosal granularity in the colon at 24 h post-challenge. Our findings indicate that CsA and Voclosporin are highly effective in swiftly alleviating oxazolone-induced inflammation, whether manifested as increased vascular permeability or fibrin accumulation at 12 h post-challenge. Notably, this inflammation was significantly reversed in the same mice by 24 h post-challenge, in contrast to the sustained inflammation observed in solvent control-treated mice.

### Voclosporin increases the expression of the sealing protein claudin 3 in the colon

**Figure 4:**
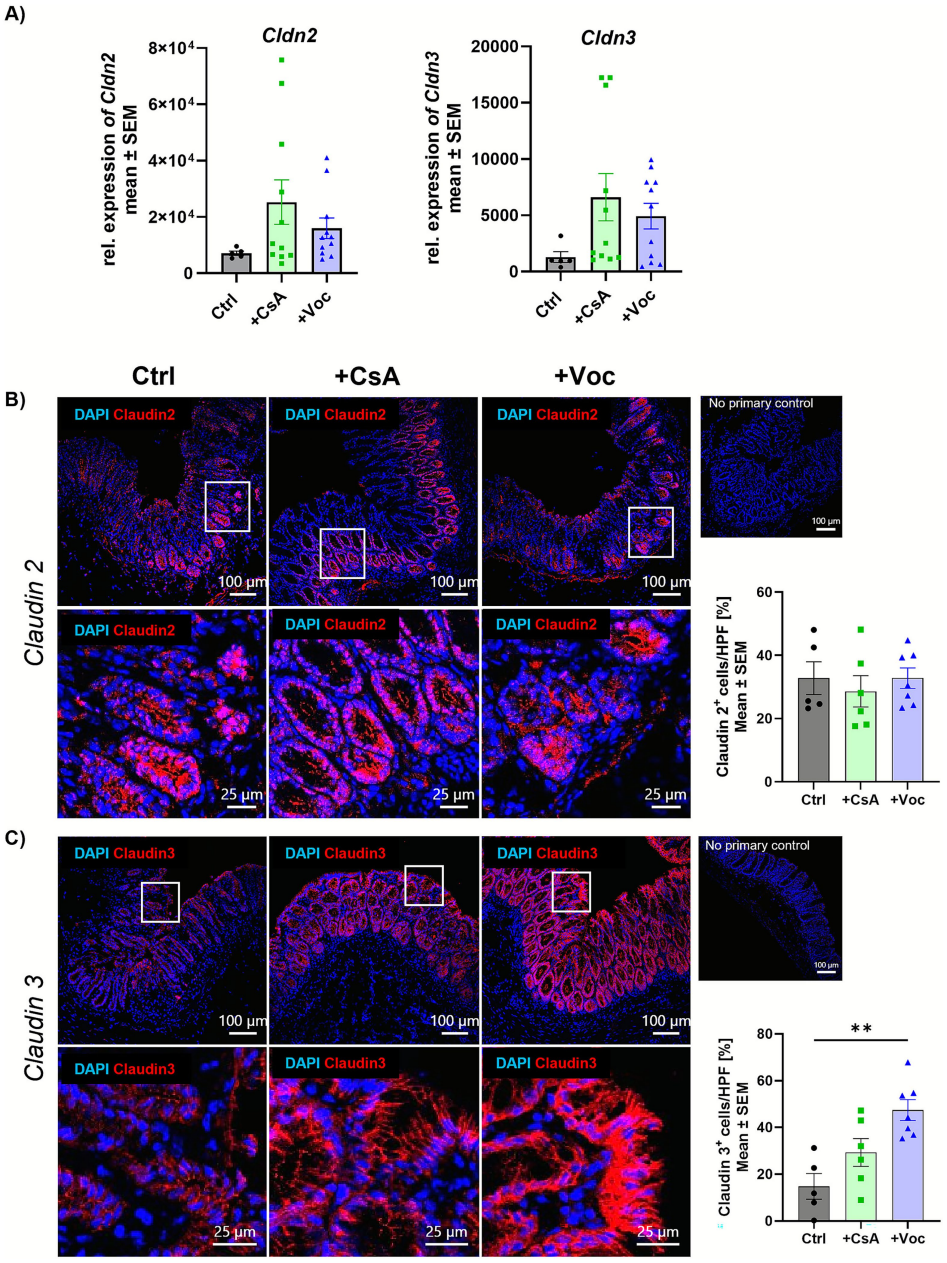
Voclosporin does not change pore forming claudin 2, but restores the sealing protein claudin 3. Acute experimental colitis was induced in mice that were further either treated with CsA or Voclo or a solvent control. Transcript expression of the *Cldn2* and *Cldn3* genes from complete murine colon tissue was determined by qPCR analysis and showed no difference between the groups **(A)** (*n* = 5–11). Cryo sections from colonic tissue slides were IF stained for claudin 2 **(B)** and claudin 3 **(C)** (*n* = 5–12). Cells were counterstained with DAPI. Mean values ± SEM of two individual experiments are shown (**p < 0.01).

Claudins represent a family of transmembrane proteins regulating intestinal barrier integrity (22). Dysregulated expression of claudins compromises barrier functions, leading to bacterial translocation and subsequent inflammation (23). Claudin 3 is recognized as a critical sealing protein in the gut, with its absence leading to hyperpermeability and exacerbation of colitis severity, whereas claudin 2 is known for its pore-forming properties (24). On transcript level no significant changes could be observed neither for claudin 2 (*Cldn2*) nor for claudin 3 (*Cldn3*) even though *Cldn* levels were increased in the calcineurin inhibitor-treated groups compared to the control group (Figure 4A). IF staining revealed in both the acute and preventive setting no alterations of claudin 2 protein expression across all groups (Figure 4B, Supplementary Figure 3). At the same time, low claudin 3 expression was detected in Ctrl mice with acute inflammation. Treatment with CsA increased claudin 3 levels to approximately ~30%. However, in mice that received Voclosporin, the number of claudin 3+ cells more than doubled compared to the control group (Figure 4C). Interestingly, both CsA and Voclosporin elevated claudin 3 expression in the colon compared to the control group in the preventive treatment setting, however, only the Voclosporin-treated group showed a significant increase of claudin 3 (Supplementary Figure 4). These findings suggest that Voclosporin significantly and, to a lesser extent, CsA can improve intestinal barrier integrity. Both calcineurin inhibitors seem to have an impact on specific signaling pathways involved in barrier reinforcement rather than permeability enhancement.

### Cyclosporine A and Voclosporin alleviate the activation of intracellular kinases in T cells

**Figure 5:**
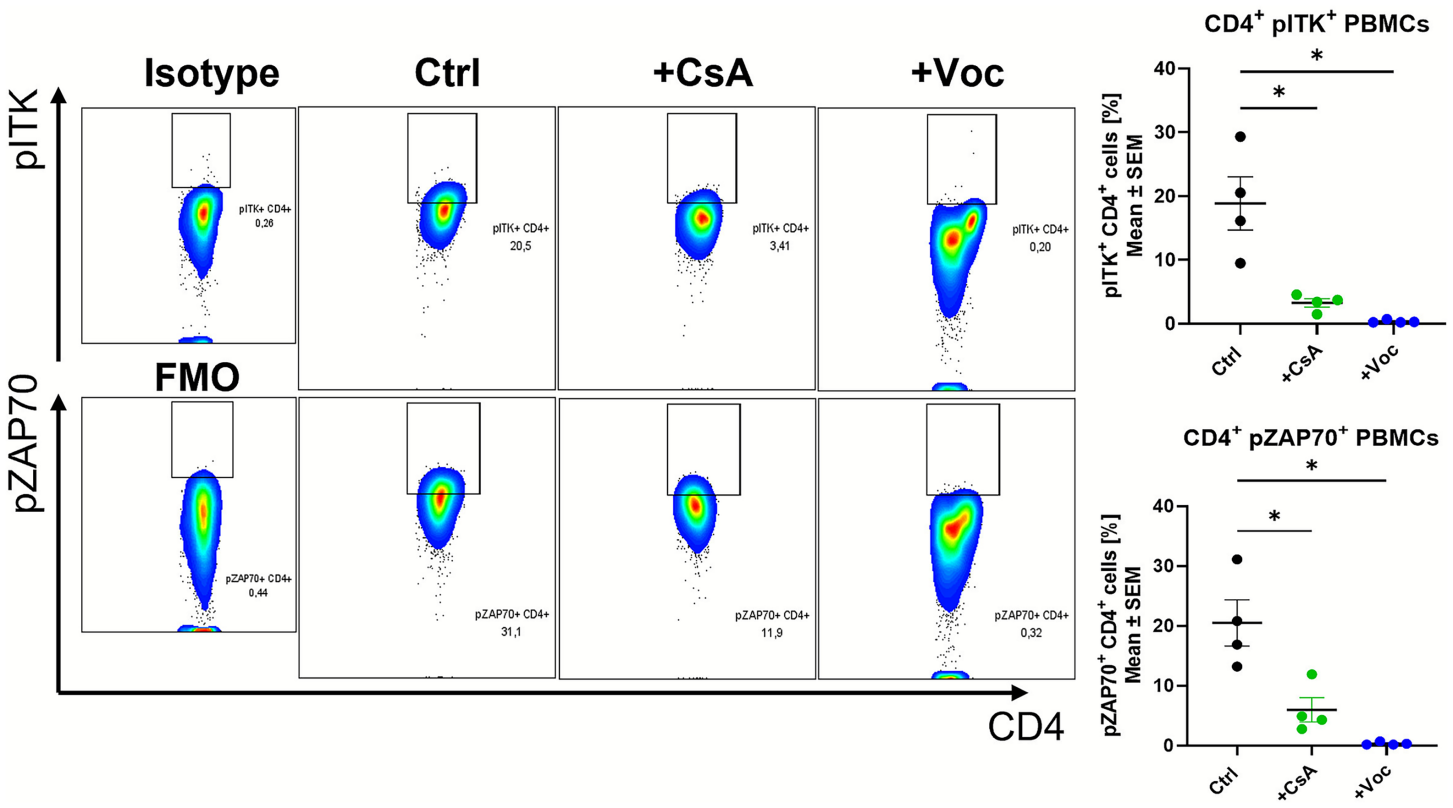
*In vitro* treatment of αCD3/αCD28 stimulated human PBMCs from healthy controls with CsA or Voc suppressed the phosphorylation of ITK and ZAP70. PBMCs from healthy controls (*n* = 4) were stimulated with αCD3 (1 g/mL), αCD28 (2 g/mL), and cultured for 24 h with solvent or CsA (10 g/mL) or Voc (10 g/mL). Afterwards, pITK and pZAP70 protein expression were measured using flow cytometry. Gates show the expression of pITK or pZAP without (Ctrl) or with the addition of CsA (+CsA) or Voc (+Voc). On the left side sotype or FMO control is shown as gating control. Mean values ± SEM are shown (*p < 0.05).

As we could show the efficacy of CsA and Voclosporin in dampening the transcript expression of the intracellular kinases Lck, Zap70 and Itk in cultured colonic LPMCs, we wanted to examine whether these pharmaceuticals are also effective in human immune cells. Therefore, we isolated PBMCs from healthy controls and stimulated them with anti-CD3/CD28 for 24 h in the presence of CsA, Voclosporin or solvent control. Flow cytometry analysis revealed that the treatment with a concentration of 10 μg/mL with both drugs could reduce the percentage of CD4+pITK+ and CD4+Zap70+ cells (Figure 5). This effect was also observed at a concentration of 25 μg/mL (Supplementary Figure 5). pITK and pZAP70 levels in CD4+ cells were reduced drastically by CsA or Voclosporin treatment, and the percentage of double-positive cells shrank to almost 0% with both concentrations. However, only Voclosporin led to elevated levels of dead cells (Supplementary Figure 6). Collectively, both pharmaceuticals caused alleviated phosphorylation of the intracellular kinases Zap70 and Itk. This shows that functionally, the capacity of CsA and Voclosporin to suppress CD4 T cell activity is associated with the phosphorylation of ITK and ZAP70.

## Discussion

The available treatment choices for IBD remain limited, with a significant portion of patients being classified as non-responders (4, 5). Voclosporin, a novel calcineurin inhibitor, presents a potential solution. It has already gained approval for the treatment of lupus nephritis, offering advantages with its oral administration (6, 8). Additionally, unlike Voclosporin’s chemical counterpart CsA, which is currently utilized in IBD patients and requires therapeutic drug monitoring, Voclosporin eliminates the need for such monitoring (7, 8). Consequently, we anticipate promising outcomes from repurposing Voclosporin for future considerations in IBD medication. In a recent study, Lindemann et al. conducted the initial comparison between Voclosporin and CsA in the context of inflammatory bowel disease (9). Employing a dextran sodium sulfate (DSS) colitis model, the researchers demonstrated that both Voclosporin and CsA effectively suppressed intestinal inflammation. The assessment included parameters such as weight loss, colon length, endoscopy, and mucosal injury in the DSS-treated animals, revealing positive outcomes with the CsA/Voclosporin therapy (9).

In order to reinforce the findings, we opted to carry out an additional experimental setting using a mouse model of colitis, which mimics more an UC-like inflammation. Specifically, we selected an acute oxazolone-induced colitis mouse model, as our aim was to employ a more immunologically T-cell-driven model. This choice allowed us to investigate the impact of CsA and Voclosporin on lymphocytes and TCR signaling in greater detail (25). Furthermore, the use of oxazolone offers advantages over the application of DSS. While oral administration of DSS gradually induces epithelial damage, subsequently leading to impaired barrier function, primary innate immune activation, and immune cell infiltration, rectal oxazolone administration results in an acute inflammation of the distal colonic mucosa and submucosa. This inflammation is characterized by ulcerations and infiltration of immune cells into the lamina propria and overall reflects a colitis predominantly driven by adaptive immune mechanisms (25). Inducing acute oxazolone colitis, we were able to demonstrate that treatment with both CsA as well as Voclosporin alleviated the severity of colitis. By employing a mechanistically distinct and complementary model, our study extends the findings from Lindemann et al. and demonstrates the robustness of Voclosporin’s immunomodulatory effects across different experimental settings. Nonetheless, we acknowledge that oxazolone colitis represents an acute inflammatory model and does not fully capture the chronic nature of ulcerative colitis, highlighting the need for additional models in future studies. We were able to demonstrate the efficacy of Voclosporin and confirm the protective effects observed in the previous DSS colitis study (9). We detected significant improvements, including reduced weight loss, significantly fewer signs of inflammation in the endoscopic imaging and HE staining in CsA- or Voclosporin-treated animals. In addition, CsA or Voclosporin treatment showed significantly reduced *in vivo* MPO activity as shown via *in vivo* IVIS imaging, indicating less severe inflammation. Furthermore, we aimed to elucidate the influence of CsA and Voclosporin on the production of cytokines by LPMCs, as they are crucial drivers of inflammation. Lechner et al. previously demonstrated that the use of CsA significantly suppresses the production of pro-inflammatory cytokines in supernatants of cultured LPMCs isolated from CsA treated mice with induced colitis (11). Similar to the effects observed with CsA, treatment with Voclosporin led to a broad reduction in cytokine production by cultured LPMCs, including IL2, IL4, IL5, IL6, IL10, IL13, IL17A, IL17F, IL22, IFNγ, and TNFα. Many of these cytokines play central roles in driving intestinal inflammation in IBD, particularly Th1- and Th17-associated mediators such as IFNγ, IL17A/F, IL22, and TNFα, which contribute to sustained immune activation and mucosal damage (26, 27). The simultaneous dampening of multiple effector pathways suggests that Voclosporin does not target a single inflammatory axis but rather broadly restrains dysregulated T-cell–mediated immune responses characteristic of active intestinal inflammation. In this context, the concurrent reduction of TNFα, a key driver of mucosal injury, and IL10, a critical regulator of immune homeostasis, likely reflects global suppression of T-cell activation rather than selective modulation of individual cytokine circuits (26, 27). While such broad immunomodulation may be beneficial during acute inflammation, the long-term consequences for regulatory immune mechanisms warrant further investigation. Previous studies have demonstrated that cyclosporine A inhibits ITK, an effect that appears central to the anti-inflammatory action of calcineurin inhibitors in colitis (11). Therefore, we examined the ITK transcript expression in the cultured LPMCs, along with the kinases ZAP70 and LCK, which are closely located to ITK and implicated in TCR signaling (28). Cultured LPMCs showed a significant suppression of both ITK and ZAP70 transcript expression following CsA or Voclosporin treatment; however, the Voclosporin treatment solely resulted in significantly reduced Lck expression levels. This suggests that both CsA and Voclosporin are capable of suppressing the transcript expression of the kinases ITK, LCK, and ZAP70, which are located in T cells and are components of the TCR signaling pathway (28). This may contribute to a reduced activity of T cells and a decreased release of proinflammatory cytokines. In summary, we assume that CsA and Voclosporin have a comparable mechanism of action.

In this study, we showed with MPEM that mice treated with CsA and Voclosporin had faster recovery of vascular permeability, accompanied by reduced vascular leakage 12 h and even more pronounced 24 h after colitis induction compared to controls. This was reflected in increased vascular permeability 12 h after treatment in response to the initial oxazolone challenge, which rapidly subsided 24 h after provocation in mice treated with inhibitors. Furthermore, Voclosporin in particular appears to have an effect on specific signaling pathways involved in barrier reinforcement rather than on permeability increase. It significantly increased the expression of the sealing protein claudin 3 in the colon in the acute situation, while the pore-forming claudin 2 remained unaffected. The preventive administration of CsA and Voclosporin prior to the induction of experimental colitis enhanced claudin 3 expression in the colon in both calcineurin inhibitor-treated groups. Taken together, this suggests that Voclosporin facilitates faster recovery of vascular permeability and leakage and contributes to the restoration of intestinal barrier integrity by increasing claudin 3 expression. While we did not directly assess barrier permeability, the observed increase in claudin 3 expression at tight junctions is consistent with its established role in maintaining intestinal barrier integrity. Loss of claudin 3 promotes gut dysbiosis and increases susceptibility to colitis (24), and reduced claudin 3 expression has been reported in IBD patients (29) as well as in TNBS- and DSS-induced colitis models (30, 31). These findings support the notion that both expression and proper localization of claudin 3 are functionally relevant for epithelial barrier maintenance.

This novel observation indicates that calcineurin inhibitors have an effect on the endothelial and epithelial cells influencing the permeability, although the complete mechanism remains unclear. The question arises whether the reduction in permeability at 12 and 24 h post colitis-induction is due to the direct effect of these drugs on endothelial cells or if it results from the mediated overall lymphocyte deactivation and cytokine production reduction, or a combination of both events.

To emblaze the treatment efficacy and effectiveness of Voclosporin and CsA in human samples we used a model of human PBMCs from healthy objects and stimulated them with αCD3/α28 antibodies in the presence or absence of CsA or Voclosporin. Lindemann et al. already showed that the application of CsA or Voclosporin in cultured human PBMCs abrogated the production of many pro-inflammatory cytokines using ELISA and qPCR. They further showed that the expression of surface activation markers is reduced in the presence of both (9). We therefore investigated the underlying mechanism of action and focused on pITK. As already demonstrated by Lechner et al., CsA is able to block the phosphorylation of ITK suggesting that the ability of CsA to suppress T cell activity is critically dependent on ITK (11). We assumed that the downstream signaling of Voclosporin works similarly, as Voclosporin is an analogon of cyclosporine A (7). When analysing CD4 + PBMCs for the phosphorylation of the intracellular kinases Zap70 and Itk, we found that both calcineurin inhibitors not only suppress the phosphorylation of ITK but also decrease the levels of pZAP70. These results align with previously mentioned murine data. A limitation of the present *in vitro* study is that Voclosporin reduced PBMC viability, which may contribute to the observed suppression of pITK/pZAP70. By contrast, CsA inhibited these phosphorylation events without affecting cell viability, indicating a specific signaling effect independent of cytotoxicity. Further, we acknowledge that ITK activation assessed in healthy donor PBMCs may not fully reflect T-cell signaling in ulcerative colitis. Healthy PBMCs were used due to availability and experimental consistency and were stimulated with anti-CD3/CD28 to mimic an activated, inflamed state. While not a perfect representation of patient-specific immune dysregulation, this approach provides mechanistic insight into ITK-dependent TCR signaling and complements our disease-relevant *in vivo* analyses.

Conclusively, Voclosporin demonstrates a protective effect in oxazolone-induced colitis and exhibits a mechanism of action similar to CsA. Notably, the use of calcineurin inhibitors also influences tissue permeability, suggesting a potential new target for resolving inflammation. Given the impressive results of Voclosporin in other diseases like lupus nephritis (6, 8) and considering its improved tolerability, repurposing this drug for conditions such as inflammatory bowel disease seems promising. This study, along with the findings of Lindemann et al., underscores the positive protective effects of Voclosporin in early murine studies of IBD (9). Further investigations and experiments are necessary to substantiate the benefits of Voclosporin in diverse indications and models.

## Materials and methods

### Mouse strains

Female C57BL/6 mice were maintained in a pathogen-free environment in the animal facility of the University Hospital of Erlangen at the Kussmaul Campus for Medical Research. Female mice were used throughout this study to ensure consistency and reproducibility of the experimental colitis model. In addition, the use of female mice reduces stress- and aggression-related confounders, thereby improving experimental robustness. The experiments were carried out with the authorization of the Middle Franconian government (approval number: 54-2532.1-15/12). Mice aged 8 to 10 weeks on a C57BL/6 background were utilized in this study.

### Experimental oxazolone-induced colitis model

Mice were randomly divided into three experimental groups, including a control group (Ctrl) receiving solvent solely, a cyclosporine A receiving group (+CsA) and a group treated with Voclosporin (+Voc). In all groups an acute colitis was induced via the hapten oxazolone (25). Four days after presensitization with 3% oxazolone (Sigma-Aldrich), mice were challenged with 0.8% oxazolone in 50% ethanol to initiate inflammation. In the acute treatment setting, intraperitoneal (IP) injections of cyclosporine A (10 mg/kg), Voclosporin (10 mg/kg), or solvent control (ethanol in PBS) were administered on the day of presensitization, the day of oxazolone challenge, and the day following the challenge. In the preventive treatment setting, cyclosporine A, Voclosporin, or solvent control was administered 6, 4, and 3 days prior to the oxazolone challenge, with an additional dose given immediately before colitis induction. Mice were sacrificed the 2nd day after inducing inflammation, and samples for subsequent analysis were taken.

### Endoscopic and *in vivo* imaging systems analyses

Twenty-four hours after challenging the mice with oxazolone, mini-endoscopy (Coloview; Storz) and *in vivo* imaging for myeloperoxidase activity (MPO) via IVIS imaging was performed (32, 33). The Murine Endoscopic Index of Colitis Severity (MEICS) was used to evaluate colitis severity at the end of the experiment (34).

### *In vivo* colonic vascular permeability imaging via multiphoton endomicroscopy (MPEM)

The analytical method used in this study was further developed from a previous publication (21) and is currently being described in more detail in a manuscript in preparation by the Division of Molecular and Experimental Surgery, University Clinic Erlangen, Germany. Changes in vascular permeability were monitored longitudinally *in vivo* in oxazolone-treated mice using MPEM. In short, 70 kDa FITC-dextran (Sigma–Aldrich) was injected into the mice at a dose of 100 mg as a contrast agent, followed by *in vivo* MPEM imaging of colonic vascularity. Images were acquired at locations in the distal colon of each mouse. Vascular leakage is identified as the escape of fluorescently labeled dextran molecules from the blood vessels into the colonic mucosal tissue. The data presented here were generated using this validated approach. In addition, the extent of FITC-dextran leakage from the vessels was quantified using ImageJ (Fiji). For this purpose, the length of the leaking vessel was determined at several points on the MPEM images (ROI length *n* = 15 per image) and numerically determined.

### LPMC cell isolation

To isolate lamina propria mononuclear cells (LPMCs) flushed colon pieces were incubated in a separation solution containing Hank’s balanced salt solution, EDTA, EGTA, and FCS. After separation and filtration, the colon pieces were transferred into a digestion solution consisting of DMEM-F12 and LPMC isolation enzymes (Miltenyi Biotec) and incubated on a magnetic stirring plate for 30 min. Subsequently, cells were collected, washed, and transferred in a 48-well plate (2.5 × 10^6^ cells/mL). The RPMI medium contained 10% fetal calf serum, 1% penicillin–streptomycin and additionally gentamycin (50 μg/mL) and amphotericin B (0.25 μg/mL). For stimulation, 1 mg/mL anti-mouse CD3 and 2 mg/mL anti-mouse CD28 antibodies were added. LPMCs were incubated for 48 h before collecting the supernatant (ELISA) and cells (qPCR).

### Measurement of cytokine concentrations

After 48 h of stimulation, LPMC supernatants were collected, centrifuged, and the secretion of cytokines was measured utilizing the LEGENDplex™ Mu Th Cytokine (Biolegend, San Diego, CA, USA). Required flow cytometry analysis was performed on an Accuri™ C6 Flow Cytometer (BD Biosciences, Franklin Lakes, NJ, USA).

### Histopathology

Hematoxylin and eosin were used to histologically stain colonic paraffin-embedded sections. To evaluate the intestinal inflammation, the following parameters were analysed: inflammatory cell infiltration, epithelial hyperplasia, mucin depletion, erosions, and ulcerations. This resulted in an additive score between 1 (none) and 5 (marked) (35, 36).

### Immunofluorescence staining

To measure MPO activity *in situ*, colonic cryosections were permeabilized, blocked with PBS containing 1% bovine serum albumin, and stained with anti-mouse MPO (R&D) and anti-goat Alexa488 (Invitrogen). Claudin staining was performed using anti-mouse claudin 3/2 (Invitrogen) with anti-rabbit Alexa555. Counterstaining was performed with Dapi. A Leica SP5 confocal microscope was used for imaging. For image adjustments and automated counting of MPO- and claudin-positive cells, ImageJ (Fiji) and QuPath were used (37, 38).

### Quantitative polymerase chain reaction

Cultured LPMCs were used for RNA isolation (NucleoSpin^®^ Macherey-Nagel). Isolated RNA was quantified using Nanodrop technology (Thermo Scientific). After reverse transcription into cDNA (iScript cDNA synthesis kit, Rio-Rad), samples were analysed using SensiFast SYBR Green (Bioline) and murine QuantiTect Primer Assays for Itk, Lck, Zap70 (Qiagen), and the housekeeping gene 18S.

### Human peripheral blood mononuclear cell (PBMC) isolation and stimulation

Blood was taken from healthy donors and a density gradient centrifugation with Pancoll (PanBiotech) was used to separate peripheral blood mononuclear cells (PBMCs). PBMCs were plated at a density of 10^6^ cells/mL in RPMI media (10% fetal calf serum, 1% penicillin–streptomycin, and 1% L-glutamine) with 1 mg/mL anti-human CD3, 2 mg/mL anti-human CD28 antibodies (Biolegend) with solvent control, 10 or 25 μg/mL Cyclosporine A or 10 or 25 μg/mL Voclosporin for 24 h. Based on the literature (9, 11), we selected 10 μg/mL of Cyclopsorine A or Voclosporin as a physiologically relevant concentration and additionally assessed 25 μg/mL for comparison. The use of human blood samples was approved by the local ethics committee (approval number: 20-426_B).

### Flow cytometry

0.5 × 10^6^ PBMCs were surface stained with anti-CD4 Pacific Blue (Biolegend) and anti-CD8 FITC (BioLegend). Zombie Aqua (Biolegend) was used as a live/dead marker. For fixation and permeabilization, a fix and perm kit (Invitrogen) was used. Intracellular staining was performed using anti-pITK PE (Invitrogen), anti-pZap70 PE/Cy7 (Biolegend). Required flow cytometry analysis was performed on a BD Symphony A1 Flow Cytometer (BD Biosciences, Franklin Lakes, NJ, USA). The gating strategy used for the Flow cytometry measurements is shown in Supplementary Figure 7.

### Statistics

Statistical differences between groups were determined using an ordinary one-way ANOVA and Dunnett’s multiple comparisons test. GraphPad Prism 10.1.2 was used for statistical analysis. *p* values < 0.05 were considered statistically significant and identified with asterisks (* < 0.05; ** < 0.01; *** < 0.001; **** < 0.0001). Results were expressed as mean values ± SEM.

## Data Availability

The raw data supporting the conclusions of this article will be made available by the authors, without undue reservation.

## Supplementary material

SUPPLEMENTARY FIGURE 1 Cyclosporine A and Voclosporin treatment ameliorate acute oxazolone-induced colitis in mice. Acute experimental colitis was induced in mice that were further either treated with CsA or Voclo or a solvent control (*n* = 5–12). Weight analysis of day 6 and 7 representing day 1 and day 2 after colitis induction. Mean values ± SEM of two individual experiments are shown (**p* < 0.05).

SUPPLEMENTARY FIGURE 2 Cyclosporine A and Voclosporin treatment ameliorate acute oxazolone-induced colitis in mice. Acute experimental colitis was induced in mice that were further either treated with CsA or Voclo or a solvent control (*n* = 4–8) in a preventive setting **(A)**. Weight analysis **(B)**, endoscopic imaging and HE staining with corresponding scoring and IF staining of MPO **(C)**. *In vivo* imaging of MPO activity using IVIS systems **(D)**. Mean values ± SEM are shown (**p* < 0.05, *****p* < 0.0001). **(A)** Created in BioRender. Knauß, A. (2026) https://BioRender.com/21ziwjz.

SUPPLEMENTARY FIGURE 3 Voclosporin and CsA do not change pore forming claudin 2. Acute experimental colitis was induced in mice that were further either treated with CsA or Voclo or a solvent control (*n* = 4–8) in a preventive setting. Cryo sections from colonic tissue slides were IF stained for claudin 2. Cells were counterstained with Dapi. Mean values ± SEM are shown.

SUPPLEMENTARY FIGURE 4 Voclosporin and CsA restore the sealing protein claudin 3. Acute experimental colitis was induced in mice that were further either treated with CsA or Voclo or a solvent control (*n* = 4–8) in a preventive setting. Cryo sections from colonic tissue slides were IF stained for claudin 3. Cells were counterstained with Dapi. Mean values ± SEM are shown (**p* < 0.05, ***p* < 0.01).

SUPPLEMENTARY FIGURE 5 *In vitro* treatment of αCD3/αCD28 stimulated human PBMCs from healthy controls with CsA or Voc suppressed the phosphorylation of ITK and ZAP70. PBMCs from healthy controls (*n* = 4) were stimulated with αCD3 (1 μg/mL), αCD28 (2 μg/mL) and cultured for 24 h with solvent or CsA (25 μg/mL) or Voc (25 μg/mL). Afterwards, pITK and pZAP70 protein expression were measured using flow cytometry. Gates show the expression of pITK or pZAP without (Ctrl) or with the addition of CsA (+CsA) or Voc (+Voc). On the left side Isotype or FMO control is shown as gating control. Mean values ± SEM of two individual experiments are shown (**p* < 0.05, ***p* < 0.01).

SUPPLEMENTARY FIGURE 6 *Live/Dead staining* of αCD3/αCD28 stimulated human PBMCs from healthy controls with CsA or Voc showed decreased number of living cells after treatment with CsA or Voc. PBMCs from healthy controls (*n* = 4) were stimulated with αCD3 (1 μg/mL), αCD28(2 μg/mL) and cultured for 24 h with solvent or CsA (10 μg/mL or 25 μg/mL) or Voc (10 μg/mL or 25 μg/mL). Afterwards, dead cells were measured using flow cytometry. Gates show the expression live cells. Mean values ± SEM are shown (**p* < 0.05, ***p* < 0.01).

SUPPLEMENTARY FIGURE 7 Gating strategy for the measurement of pITK or pZAP70 in CD4+ PBMCs. All Events were discriminated for single cells. Afterwards gates were set according to lymphocyte population. After excluding dead cells, CD4+ and CD8+ cells were differentiated. The next gate is used to detect pITK or pZAP+ CD4+ cells according to isotype or FMO control.
