# Supplementary figures and images for: Voclosporin shows protective effect and intestinal barrier enforcement in experimental colitis
Source: Front Med (Lausanne). 2026 Feb 2;13:1750826. doi: 10.3389/fmed.2026.1750826 (PMC12908035; doi:10.3389/fmed.2026.1750826)

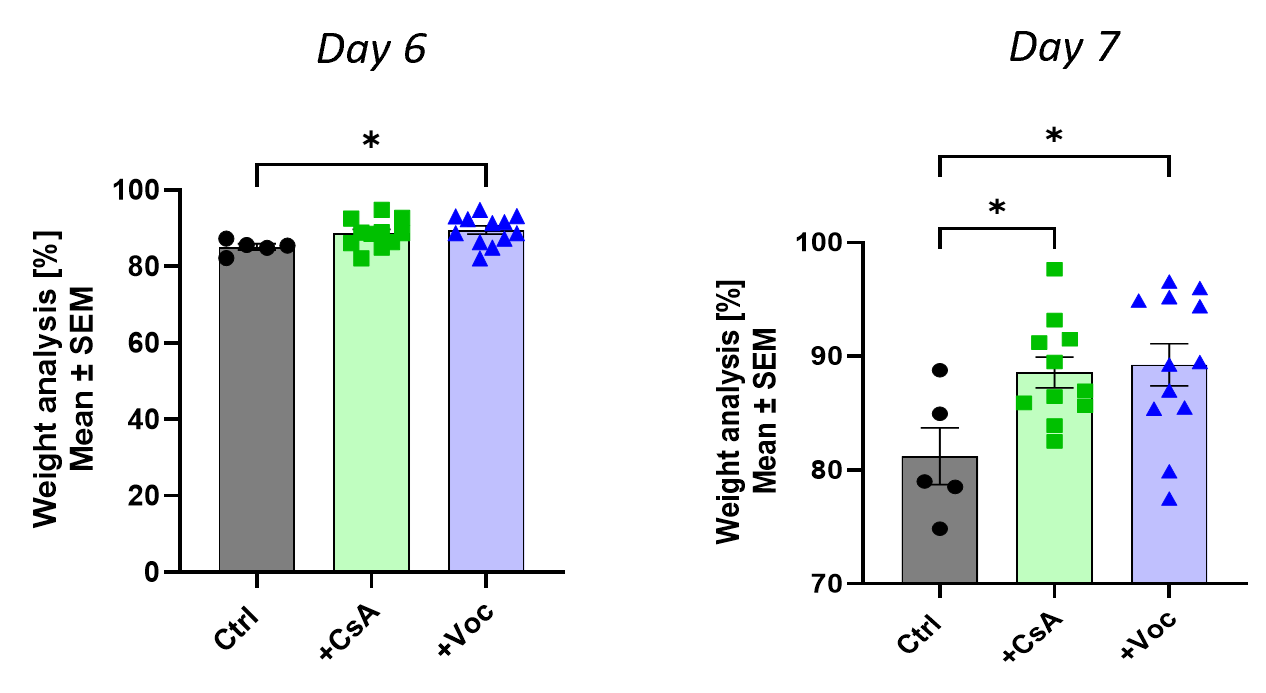

Supplement: SUPPLEMENTARY FIGURE 1 — Cyclosporine A and Voclosporin treatment ameliorate acute oxazolone-induced colitis in mice. Acute experimental colitis was induced in mice that were further either treated with CsA or Voclo or a solvent control (n = 5–12). Weight analysis of day 6 and 7 representing day 1 and day 2 after colitis induction. Mean values ± SEM of two individual experiments are shown (*p < 0.05). [file Image_1.tif]

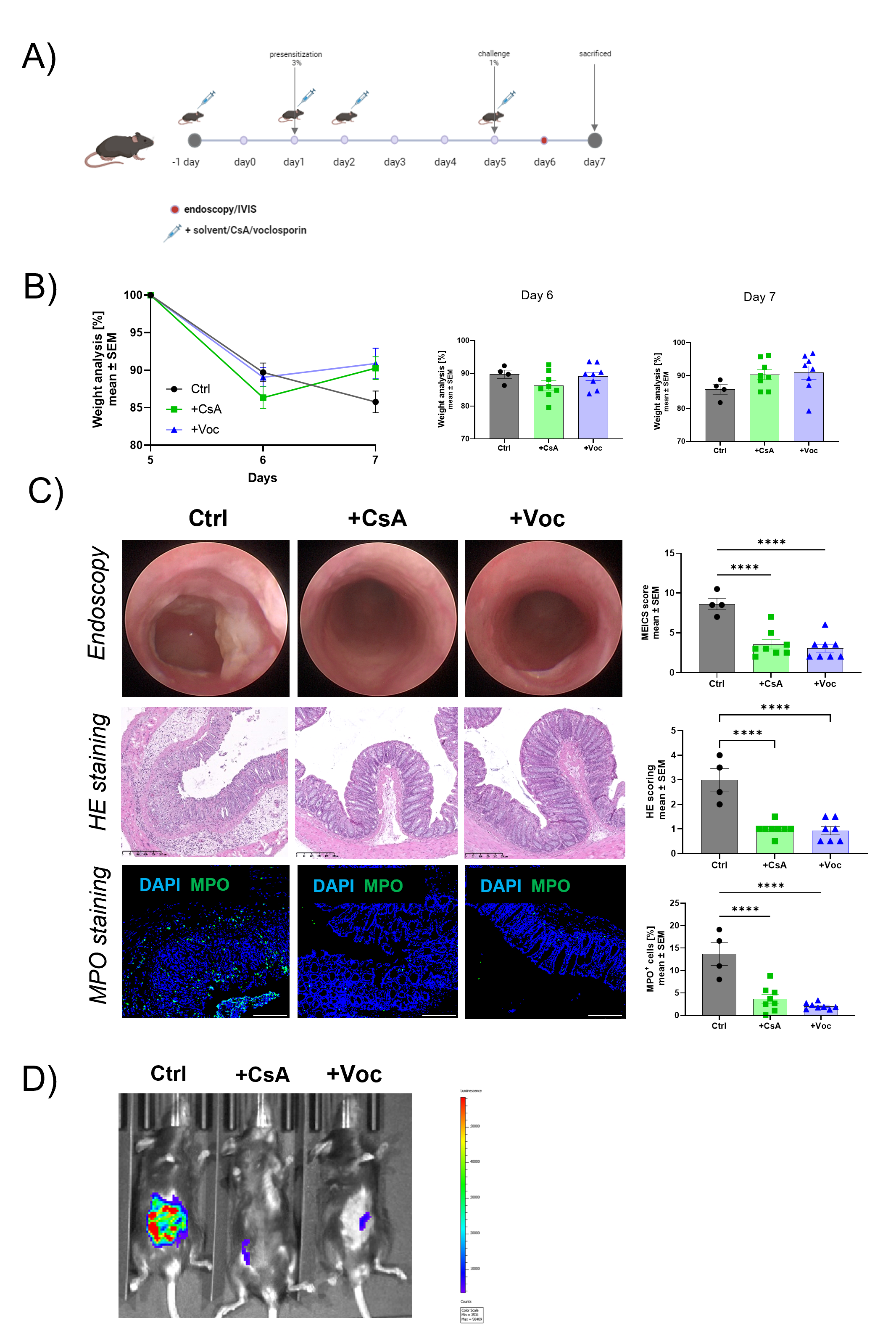

Supplement: SUPPLEMENTARY FIGURE 2 — Cyclosporine A and Voclosporin treatment ameliorate acute oxazolone-induced colitis in mice. Acute experimental colitis was induced in mice that were further either treated with CsA or Voclo or a solvent control (n = 4–8) in a preventive setting (A). Weight analysis (B), endoscopic imaging and HE staining with corresponding scoring and IF staining of MPO (C). In vivo imaging of MPO activity using IVIS systems (D). Mean values ± SEM are shown (*p < 0.05, ****p < 0.0001). (A) Created in BioRender. Knauß, A. (2026) https://BioRender.com/21ziwjz. [file Image_2.tif]

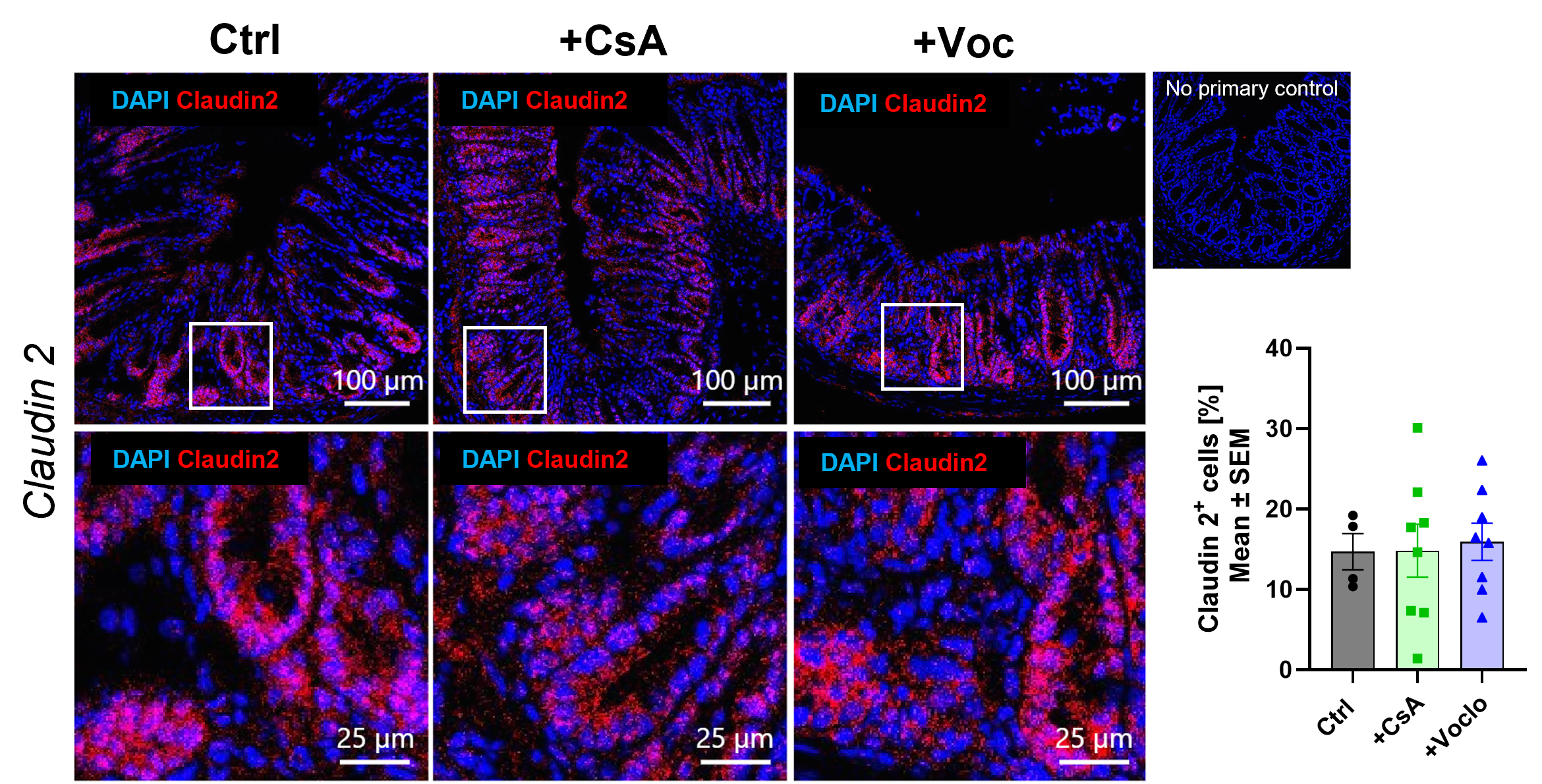

Supplement: SUPPLEMENTARY FIGURE 3 — Voclosporin and CsA do not change pore forming claudin 2. Acute experimental colitis was induced in mice that were further either treated with CsA or Voclo or a solvent control (n = 4–8) in a preventive setting. Cryo sections from colonic tissue slides were IF stained for claudin 2. Cells were counterstained with Dapi. Mean values ± SEM are shown. [file Image_3.tif]

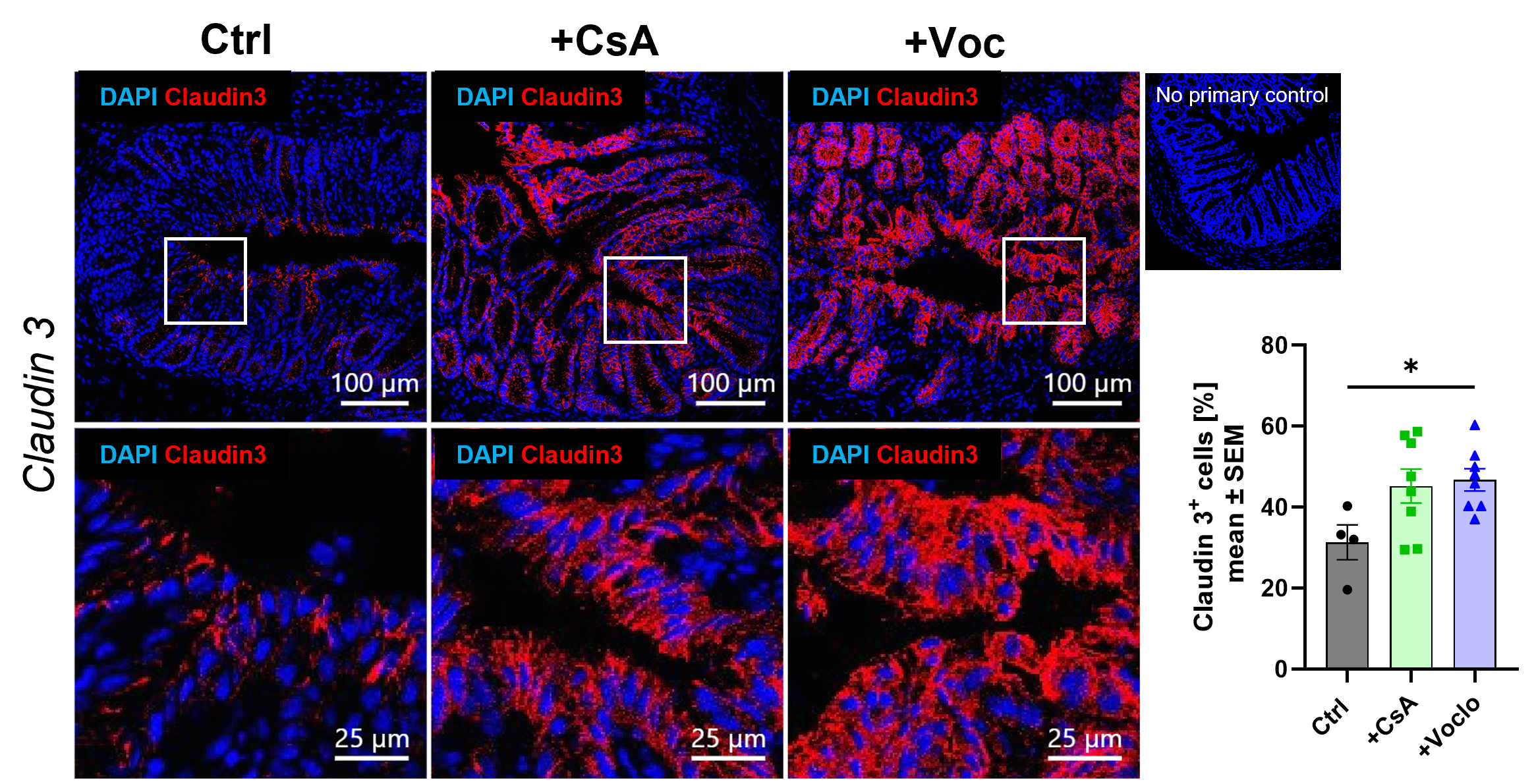

Supplement: SUPPLEMENTARY FIGURE 4 — Voclosporin and CsA restore the sealing protein claudin 3. Acute experimental colitis was induced in mice that were further either treated with CsA or Voclo or a solvent control (n = 4–8) in a preventive setting. Cryo sections from colonic tissue slides were IF stained for claudin 3. Cells were counterstained with Dapi. Mean values ± SEM are shown (*p < 0.05, **p < 0.01). [file Image_4.tif]

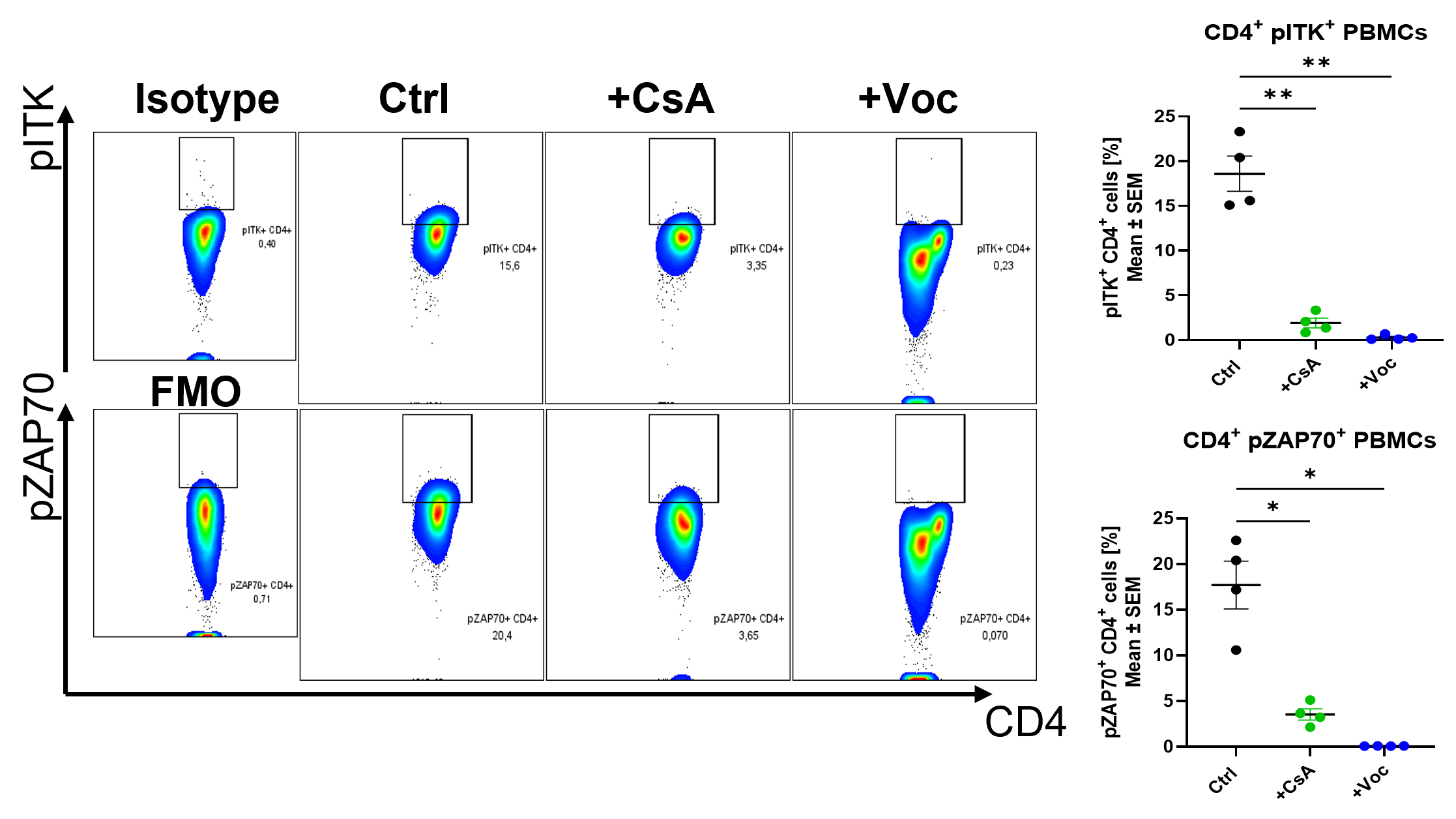

Supplement: SUPPLEMENTARY FIGURE 5 — In vitro treatment of αCD3/αCD28 stimulated human PBMCs from healthy controls with CsA or Voc suppressed the phosphorylation of ITK and ZAP70. PBMCs from healthy controls (n = 4) were stimulated with αCD3 (1 μg/mL), αCD28 (2 μg/mL) and cultured for 24 h with solvent or CsA (25 μg/mL) or Voc (25 μg/mL). Afterwards, pITK and pZAP70 protein expression were measured using flow cytometry. Gates show the expression of pITK or pZAP without (Ctrl) or with the addition of CsA (+CsA) or Voc (+Voc). On the left side Isotype or FMO control is shown as gating control. Mean values ± SEM of two individual experiments are shown (*p < 0.05, **p < 0.01). [file Image_5.tif]

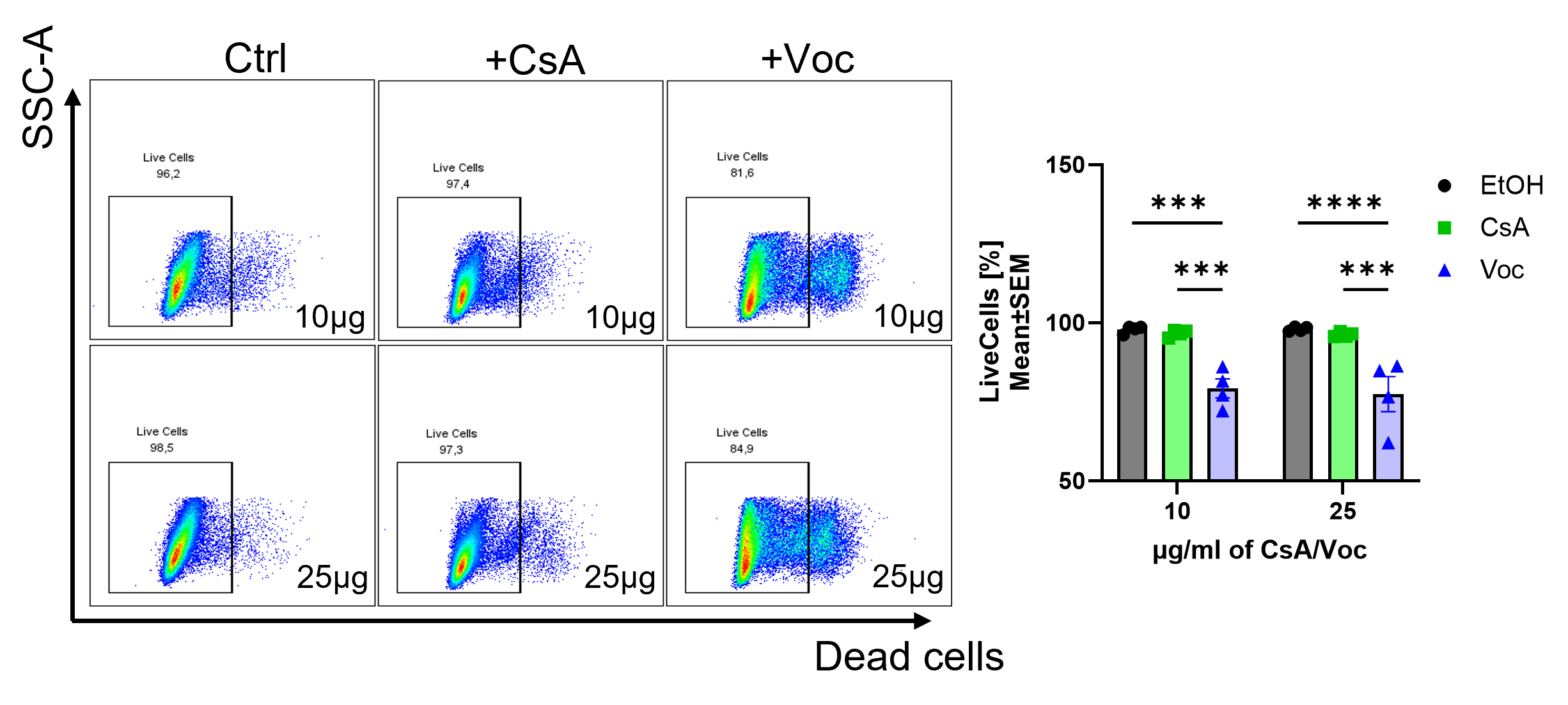

Supplement: SUPPLEMENTARY FIGURE 6 — Live/Dead staining of αCD3/αCD28 stimulated human PBMCs from healthy controls with CsA or Voc showed decreased number of living cells after treatment with CsA or Voc. PBMCs from healthy controls (n = 4) were stimulated with αCD3 (1 μg/mL), αCD28(2 μg/mL) and cultured for 24 h with solvent or CsA (10 μg/mL or 25 μg/mL) or Voc (10 μg/mL or 25 μg/mL). Afterwards, dead cells were measured using flow cytometry. Gates show the expression live cells. Mean values ± SEM are shown (*p < 0.05, **p < 0.01). [file Image_6.tif]

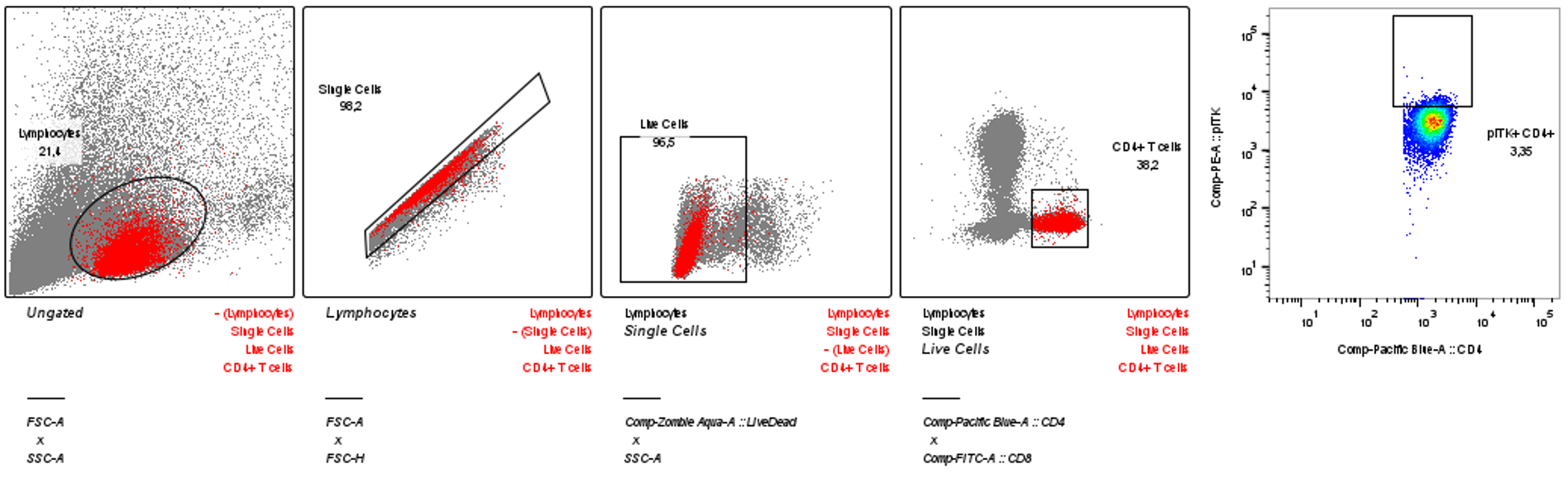

Supplement: SUPPLEMENTARY FIGURE 7 — Gating strategy for the measurement of pITK or pZAP70 in CD4+ PBMCs. All Events were discriminated for single cells. Afterwards gates were set according to lymphocyte population. After excluding dead cells, CD4+ and CD8+ cells were differentiated. The next gate is used to detect pITK or pZAP+ CD4+ cells according to isotype or FMO control. [file Image_7.tif]
